# Clinical Development of Targeted Fragile X Syndrome Treatments: An Industry Perspective

**DOI:** 10.3390/brainsci8120214

**Published:** 2018-12-05

**Authors:** Anna W. Lee, Pamela Ventola, Dejan Budimirovic, Elizabeth Berry-Kravis, Jeannie Visootsak

**Affiliations:** 1 Ovid Therapeutics Inc., New York, NY 10036, USA; jvisootsak@ovidrx.com; 2 Child Study Center, Yale University, New Haven, CT 06520, USA; pamela.ventola@yale.edu; 3 Departments of Psychiatry and Behavioral Sciences, Kennedy Krieger Institute and Child Psychiatry, Johns Hopkins University, Baltimore, MD 21205, USA; budimirovic@kennedykrieger.org; 4 Departments of Pediatrics, Neurological Sciences, Biochemistry, Rush University Medical Center, Chicago, IL 60612, USA; Elizabeth_Berry-Kravis@rush.edu

**Keywords:** fragile X syndrome, clinical trials, targeted treatments, drug development

## Abstract

Fragile X syndrome (FXS) is the leading known cause of inherited intellectual disability and autism spectrum disorder. It is caused by a mutation of the fragile X mental retardation 1 (*FMR1*) gene, resulting in a deficit of fragile X mental retardation protein (FMRP). The clinical presentation of FXS is variable, and is typically associated with developmental delays, intellectual disability, a wide range of behavioral issues, and certain identifying physical features. Over the past 25 years, researchers have worked to understand the complex relationship between FMRP deficiency and the symptoms of FXS and, in the process, have identified several potential targeted therapeutics, some of which have been tested in clinical trials. Whereas most of the basic research to date has been led by experts at academic institutions, the pharmaceutical industry is becoming increasingly involved with not only the scientific community, but also with patient advocacy organizations, as more promising pharmacological agents are moving into the clinical stages of development. The objective of this review is to provide an industry perspective on the ongoing development of mechanism-based treatments for FXS, including identification of challenges and recommendations for future clinical trials.

## 1. Introduction

Fragile X syndrome (FXS) is the most common inherited cause of intellectual disability and the second most common cause of intellectual disability after Down syndrome [1,2]. It is also the most common known monogenetic cause of autism spectrum disorder (ASD), with approximately 30% to 54% of males and 16% to 20% of females with FXS meeting the diagnostic criteria for ASD by direct assessment [3]. FXS is a genetic disorder caused by the expansion of over 200 cytosine–guanine–guanine (CGG) triplet repeats in the fragile X mental retardation 1 (*FMR1)* gene on the X chromosome [4]. The normal range of CGG repeats varies from 5 to 44 [4]. The full mutation, defined as over 200 CGG repeats, results in the hypermethylation and silencing of the promoter region of the *FMR1* gene, and the absence or reduction of fragile X mental retardation protein (FMRP). FMRP is an RNA-binding protein, which regulates the synthesis of many synaptic proteins. FMRP is required for normal neural development and its absence leads to abnormalities in brain development and function [5].

Individuals with 55–200 CGG repeats in the *FMR1* gene are said to be premutation carriers [4]. In contrast to full mutation, premutation is associated with a functional gene that transcribes the mutation and is associated with certain clinical features that do not occur in those with the full mutation. Males with premutation may develop fragile X-associated tremor/ataxia syndrome (FXTAS), an adult onset neurodegenerative disorder. This phenotype usually manifests late in life (early 60s) and is characterized by action tremor, gait ataxia, and impaired cognition. Parkinsonism-like symptoms, lower extremity neuropathy, dysautonomia, anxiety and depression, and behavioral problems may also be present. Females also may develop FXTAS, in about 10% of cases [6], and symptoms tend to be milder [4]. Females with the premutation can also be affected by fragile X-associated primary ovarian insufficiency (FXPOI) [6].

*FMR1* genes with CGG repeat lengths of 45–54 are classified as intermediate or grey zone alleles. It is not yet clear whether these small expansions are related to an increased risk of disease [4]. Recent reports suggest that intermediate alleles may be associated with the development of mild FXTAS, FXPOI, Parkinson disease, ataxia, or multiple system atrophy; however, more research is needed for a clarification of definite risks. Many intermediate alleles are stable and do not change over generations. Individuals with intermediate alleles are not at risk for FXS or to have children with FXS. However, in some families, intermediate alleles with zero or one AGG interspersions can show instability and can expand to a premutation in future generations.

The clinical phenotype of FXS is more varied than basic descriptions suggest. Some individuals express the full mutation in some cells and premutation in others [6]. This “size mosaicism” can result in a milder and even mixed clinical picture, with the presence of both FXS and FXTAS having been described in a very small number of cases of older individuals with FXS. A complicated clinical picture may also be present in individuals with “methylation mosaicism”, which occurs when only some of the full mutation alleles are methylated. Furthermore, some individuals may have deletions or point mutations [7] in *FMR1* rather than—or in addition to—repeat expansion [6]. Clinical presentation in such individuals may or may not be typical.

There have been a number of studies aimed at determining the prevalence of FXS in males and females. FXS affects all ethnic and socioeconomic backgrounds. Current estimates suggest that it affects between 1 in 4000 to 5000 males and 1 in 6000 to 8000 females worldwide [1]. The prevalence of premutation carriers is more common, affecting an estimated 1 in 300 to 450 males and 1 in 150 to 200 females. These estimates translate to approximately 430,000 male and close to 1 million female premutation carriers in the US.

## 2. FXS Phenotypes

Individuals with FXS present with a broad range of physical features, symptoms, and limitations (Table 1) [4,8,9,10]. The subtle physical features and variability in the clinical presentation of FXS make diagnosis dependent on molecular confirmation. Individuals with FXS can have characteristic physical features that become more apparent with increasing age [8]. These features include a long, narrow face, tall forehead, large prominent ears, high arched palate, unusually hyperflexible fingers, and flat feet. Males also have macro-orchidism after puberty. However, in some individuals with FXS, there may be minimal or no obvious physical features.

Physical features of FXS become more apparent with increasing age [10]. Physical features are often not apparent in younger individuals [8,10]. The newborn may appear entirely normal and the only indicator of FXS in infancy may be some degree of reduced muscle tone. At this age, feeding problems with gastroesophageal refluxes (often resulting in emesis) and poor latch or suck with breastfeeding, as well as chronic otitis media are relatively common [10]. Delays in developmental and speech milestones usually become evident before 2 years of age and are the most common symptom leading to diagnosis in patients with FXS. Motor delays and variable hypotonia are present in some infants with FXS, with delays in crawling and walking. Hypotonia and motor coordination deficits become less evident as children become older. Behavioral symptoms become evident in early childhood. In older patients, the facial phenotype may become more accentuated, facilitating clinical diagnosis, but this is not uniform. Joint laxity becomes less prominent after childhood, but for many patients persists throughout life.

There is a wide range of behavioral symptoms in FXS (Table 1), although these tend to be more severe in males than in females [10]. Individuals with FXS can experience problems in one or more behavioral domains. Many individuals with FXS have features of ASD; 30% to 54% of males and 16% to 20% of females meet diagnostic criteria for ASD [3]. Early language and motor milestones [11], and language–communication and social interaction (reviewed in Budimirovic and Kaufmann, 2011) are more affected in patients with ASD and FXS than in those with FXS only. Anxiety is one of the most common reported behavioral symptoms [12,13], with specific symptoms of shyness and social anxiety especially common as patients go into adolescence. Hyperarousal is common at younger ages in particular, and stereotypic behavior such as hand flapping and hand biting are often seen. Many of these behaviors are thought to result from a high underlying level of social anxiety, poor flexibility in response to unexpected situations, and general over-responsiveness to sensory stimuli. Social anxiety in individuals with FXS can be a significant issue for families, interfering with daily activities, education, and socialization, and it may be associated to some extent with high parenting stress [14,15]. Sleep issues are commonly reported by parents of children with FXS as well. Children with FXS may experience more problems falling asleep, frequent nighttime awakenings, early awakenings, restless sleep, and daytime sleepiness than typically developing children [16].

Females with FXS usually present with less severe symptoms compared to males [6]. In some cases, females with FXS do not appear to display any obvious symptoms. This likely reflects the presence of the normal *FMR1* allele on the active X chromosome in a proportion of cells in females. Most males with FXS have moderate to severe intellectual disabilities, while only about a quarter of females with FXS have intellectual disability (an IQ < 70) [17]. Adult males have an average IQ of about 40 and a mental age of about 5–6 years [18], whereas most females typically have cognitive abilities in the borderline to low–normal range (25% IQ < 70; 28% IQ 70–84) [19]. However, there is large variability in cognitive ability, in both males and females, due to a number of factors yet to be fully understood. Additionally, IQ scores decline with age, with adolescents and adults consistently scoring lower than young children [20]. Floor effects further complicate the interpretation of test results [21]. However, individuals with FXS do not regress in their cognitive functioning; rather, they do not progress at the same rate as their peers, resulting in the lower cognitive scores over time. It is estimated that approximately 60% to 75% of females and nearly 100% of males with FXS have significant language deficits [6]. Females also are prone to the development of emotional problems, specifically internalizing disorders, such as social anxiety and depression [8].

## 3. Current State of FXS Treatment

Currently, there is no FDA-approved treatment for FXS. The associated (as opposed to the core) symptoms of FXS are typically managed using pharmacologic interventions, such as stimulants for attention deficit and hyperactivity, selective serotonin reuptake inhibitors (SSRIs) for anxiety, antipsychotic drugs for aggression and mood instability, and melatonin for sleep [22,23]. These pharmacologic treatments target only the behavioral symptoms and not the cognitive/language impairments or the underlying brain deficits. Interventional services such as speech–language therapy, occupational therapy, physical therapy, special education services, and behavior management are commonly utilized to address specific behaviors and developmental issues, and comorbid conditions such as ASD, anxiety, attention deficit/hyperactivity disorder (ADHD), seizures, frequent otitis media, strabismus, sleep difficulties, and gastrointestinal problems, which may require specialized medical care [9,22,24]. Even with substantial support and therapies, individuals with FXS continue to present with significant impairments in their functioning throughout their life.

In recent years, an effort to improve the lives of individuals with FXS has driven increased research into the pathophysiologic causes of FXS manifestations and new therapeutic approaches to manage them. The results of these studies have not only demonstrated the complexity of the relationship between FMRP deficiency, downstream neurobiological abnormalities, and FXS symptoms, but also have identified a vast array of potential interventional targets.

## 4. Development of Targeted Therapies

### 4.1. Preclinical Rationale

The relationship between FMRP deficiency and aberrant synaptic function is thought to underlie symptoms of FXS and has been the focus of intense research, using animal models of FXS. FMRP is widely expressed in mammals, particularly in the brain and gonads [25,26,27]. A primary function of FMRP appears to be the regulation of mRNA translation and the synthesis of proteins essential for normal dendritic spine morphology and neuronal signaling [5], as demonstrated by widespread structural and functional abnormalities in its absence [28,29,30]. In the brain, altered signaling when FMRP is absent is believed to be a result of excessive ribosomal activity and protein synthesis, causing a shift in the excitatory/inhibitory balance towards excessive excitation [31]. Considerable research has focused on the effects of excess group I metabotropic glutamate receptor (mGluR) signaling (excitatory), in particular mGluR5 [32] and deficient γ-aminobutyric acid (GABA) signaling (inhibitory) [33]. Research into these abnormalities and related/downstream effects has resulted in the identification of a large number of potential therapeutic targets, with the most data having been collected on effects of mGluR5 antagonists (2-methyl-6-(phenylethynyl)pyridine (MPEP), fenobam, 2-chloro-4-((2,5-dimethyl-1-(4-(trifluoromethoxy)phenyl)-1H-imidazol-4-yl)ethynyl)pyridine (CTEP), AFQ056, STX107, RO4917526), and GABA receptor activators (baclofen, arbaclofen, acamprosate, ganaxolone, gaboxadol, metadoxine) [10,18]. Other mechanisms targeted for FXS include the inhibition of pathways downstream of group I mGluR signaling (lithium, serine/threonine-protein kinase (PAK) inhibitors, lovastatin, glycogen synthase kinase-3β (GSK3β) inhibitors, PI3K enhancer (PIKE) inhibitors, P13K inhibitors, ERK/Akt inhibitors), blocking excess activity of overproduced proteins (minocycline, striatum-enriched protein-tyrosine phosphatase (STEP) inhibitors, rolipram, phosphodiesterase (PDE) inhibitors); increasing deficient α-amino-3-hydroxy-5-methyl-4-isoxazolepropionic acid (AMPA) receptor activity (CX516); blocking excess synthesis of specific proteins with micro RNAs (miR125A); regulating abnormal channel activity (BK channel blockers, KCNQ1 (Slack) channel blockers); regulating abnormal insulin signaling (metformin); inhibition of endocannabinoid signaling (cannabidiol, endocannabinoid blockers); and inhibition of acetyl cholinesterase (donepezil).

### 4.2. Clinical Development

FXS has been at the forefront of efforts to test preclinical evidence for targeted interventions in clinical studies. However, to date, the clinical translation of new FXS-specific agents has failed to meet primary endpoints in trials. For example, recent notable compounds demonstrating great preclinical promise, but failing to move forward after early phase 2 trials in the clinic include the selective GABA_B_ agonist arbaclofen (STX209 (Seaside)), the selective GABA_A_ modulator ganaxolone, the monoamine-independent GABA transmission modulator metadoxine, and the noncompetitive mGluR5 antagonists mavoglurant (AFQ056 (Novartis)) and basimglurant (RO4917523 (Roche)) [34]. It is unclear whether the failure of these compounds to show benefit in clinical trials reflects the inadequacy of the compounds themselves or the design of the studies devised to evaluate them. Some of these studies were phase 2 studies (ganaxolone, metadoxine) or closed without full enrollment due to financial issues (arbaclofen) and, thus, were statistically underpowered to detect between-group treatment differences at expected effect sizes [18], but most also relied on outcome measures that were not well matched to the expected effects of the drug and/or inadequate to detect meaningful improvements in FXS populations tested. Some studies which showed potential beneficial effects of the drug have simply not moved forward due to the financial position of the sponsor (arbaclofen, metadoxine). With respect to the latter, the Outcome Measures Working Groups convened by the National Institutes of Health to examine endpoints used in FXS clinical trials identified a number of shortcomings, including the dearth of measures validated for FXS, the failure of investigators to use a common set of measures (no consensus), the inability of available measures to assess a broad range of function, limited standardization and floor effects, and the absence of direct-observation measures or validated biological markers (biomarkers) [34].

Despite the history of several “negative” FXS trials, the search for effective FXS-targeted therapies continues. As of October 2018, ClinicalTrials.gov listed 71 FXS studies in various stages of activity, including those completed [35]. While the majority of currently active studies (planned, recruiting/enrolling, or active; Table 2) are investigator-initiated trials, the pharmaceutical industry is visibly involved in the development of at least four promising targeted agents (OV101/gaboxadol, ZYN002/cannabidiol, BPN14770, and Bryostatin-1).

#### 4.2.1. BPN14770

BPN14770 (Tetra Discovery Partners Inc., Grand Rapids, MI, USA) is a phosphodiesterase-4D negative allosteric modulator (PDE4D-NAM) [36]. It has been proposed that inhibition of PDE4 can prevent the degradation of cAMP, which is reduced in the presence of FMRP deficiency [37,38,39]. Inhibition of PDE4 may attenuate associated effects, as demonstrated by the rescue of behavioral and structural deficits in the *Drosophila* FXS model [40,41]. In a preclinical study conducted in adult male *Fmr1* KO mice, BPN14770 significantly improved behavioral phenotypes (reduced hyperarousal in the open field, increased frequency of social interaction, and improved natural behaviors (nesting and marble burying)) vs. vehicle administration, with effects persisting after a 2-week drug washout period [36]. In humanized PDE4D mice, single doses of BPN14770 increased brain cAMP, augmented the extended phase of long-term potentiation (LTP), reversed scopolamine-induced short-term memory impairment, and improved long-term memory [42].

The safety and tolerability of BPN14770 were established in two phase 1 studies conducted in healthy young and elderly subjects [43]. A phase 2 two-period crossover study (NCT03569631; target N, 30) is underway to assess the safety and tolerability of BPN14770 in individuals with FXS and explore the effects of BPN14770 on cognitive function and behavior in adult males with FXS [35]. Planned outcome assessment measures include the NIH-Toolbox cognitive battery (NIH-TCB) modified for intellectual disabilities; the KiTAP test of attentional performance; the clinical global impression–severity (CGI–S); the CGI–I; a visual analog rating scale assessment of behavior problems, language abilities, and eating behavior; the aberrant behavior checklist community edition (ABC–C); the anxiety, depression, and mood scale (ADAMS); the Vineland-3 adaptive behavior scale; electroencephalogram assessment of event-related potentials; and eye tracking.

#### 4.2.2. OV101/Gaboxadol

OV101 (gaboxadol or 4,5,6,7-tetrahydroisoxazolo-[5,4-c]pyridine-3-ol (THIP); Ovid Therapeutics Inc.) is a highly selective GABA receptor agonist. [44]. In vitro binding studies have shown that OV101/gaboxadol binds selectively to the extrasynaptic α4δ-containing GABA_A_ receptor subpopulation [44,45,46]. In contrast to OV101/gaboxadol, benzodiazepines or other agents acting at the benzodiazepine binding sites do not act on these same receptors [47]. OV101/gaboxadol, through its actions on extrasynaptic α4δ-containing GABA_A_ receptors, is believed to restore tonic inhibition and contribute to sleep induction and maintenance.

The therapeutic potential of OV101 and other GABA agonists has been tested in genetically engineered animal models of FXS that recapitulate the human phenotype of FXS. In the *Fmr1* knockout (KO) mouse model [10], FMRP deficiency is thought to lead to reductions in GABA_A_ receptors and enzymes necessary for GABA production [48] as well as defects in phasic (synaptic) and tonic (extrasynaptic) inhibitory signaling [10]. Disruptions to GABA signaling are associated with excessive neural excitation [33], a common feature of FXS. In animal models, compounds targeting the GABA_A_ receptor (agonists) were shown to improve behavioral characteristics, such as hyperactivity, auditory/audiogenic seizures, and repetitive and/or perseverative behaviors [10]. A transient induction of the excitatory–inhibitory switch was also shown to improve hyperactivity and autistic behaviors in offspring in the *Fmr1* KO mouse model. In the *Fmr1* KO, OV101 reduced sensory hypersensitivity and motor hyperactivity improved prepulse inhibition (signal to noise ratio) and enhanced tonic inhibition in the amygdala, a region of the brain thought to be associated with behavioral abnormalities in individuals with FXS [49,50]. In a separate study conducted in *Fmr1* KO mice, OV101 normalized behavioral abnormalities relevant to hyperactivity, anxiety, irritability/aggression, and repetitive behavior [51].

In a phase 1 PK clinical trial, OV101 was well tolerated in adolescents age 13–17 years with Angelman syndrome or FXS (*n* = 12) (NCT03109756) [35]. A phase 2 three-arm, double-blind clinical study (ROCKET; NCT03697161) is ongoing [35]. ROCKET is enrolling adolescent and young adult males with FXS aged 13 to 22 years. It was designed primarily to evaluate the safety of OV101 in FXS, and also includes secondary measures to assess changes in behavior and functioning using the aberrant behavior checklist community edition (ABC–C) and the clinical global impressions–improvement (CGI–I) scale.

#### 4.2.3. ZYN002

ZYN002 (Zynerba Pharmaceuticals, Inc.) is a transdermal cannabidiol gel formulation that targets the dysregulation of the endocannabinoid system [52]. Cannabidiol is a non-intoxicating cannabinoid with numerous molecular targets [53,54]. It has been reported to potentially demonstrate a broad range of activity, including analgesic, anti-inflammatory, antioxidant, antiemetic, antianxiety, antipsychotic, anticonvulsant, and selective cytotoxic effects [53,55] and has shown efficacy as an antispasticity and antiepileptic agent. It is proposed that transdermal administration will reduce the risk for psychomimetic adverse effects versus oral administration by circumventing the conversion to psychoactive components (i.e., Δ-9 tetrahydrocannabinol (THC), Δ-8 THC) in the acidic environment of the gastrointestinal tract [56].

Preclinical evidence indicates that FMRP deficiency enhances mGluR1-dependent endocannabinoid mobilization and subsequent synaptic effects [57,58,59]. This potentiates inhibitory short- and long-term depression and excitatory postsynaptic potential (EPSP)-spike coupling in the hippocampus [57]. The effects of endocannabinoid modulators in the *Fmr1* KO have been complex, with both activators and blockers of endocannabinoid activity showing phenotype reversal in different brain areas and neural cell types. A blockade of presynaptic cannabinoid type 1 receptors (CB1) normalized several FXS phenotypes in the mouse model [58], as did a blockade of the degradation of the predominant endocannabinoid, 2-arachidonoylglycerol (2-AG) [60]. A blockade of the degradation of the endocannabinoid anandamide also improved performance on tests of learning and memory in the mouse model [61].

ZYN002 was demonstrated to be safe and well tolerated in phase 1 studies conducted in healthy subjects and patients with epilepsy [62,63]. It was also well tolerated in a phase 2 open-label study and extension conducted in 20 children and adolescents with FXS, with the most common treatment-emergent adverse events being gastroenteritis and upper respiratory tract infection [52]. Improvements in behavioral symptoms were noted and are being further evaluated in an ongoing phase 3 double-blind placebo-controlled clinical trial (CONNECT-FX; NCT03614663) [35]. CONNECT-FX plans to utilize the ABC–C with the Fragile X Factor Structure and the CGI–I scale to evaluate drug effects [35].

#### 4.2.4. AFQ056

AFQ056 is an mGluR5 negative modulator (NAM). This class of drugs has shown reversal of a comprehensive list of synaptic, molecular, electrophysiological, morphological, behavioral, and cognitive phenotypes in the fragile X mouse, fly, and rat models, with consistent results from four different mGluR5 NAMs (including AFQ056) published in over 50 scientific papers. Six phenotypes in multiple categories in the fragile X mouse are also corrected by genetic reduction of mGluR5 receptors. The preclinical body of evidence surrounding this treatment target is certainly the largest in any genetic neurodevelopmental disorder [18,64]. A phase 1 trial of fenobam, another mGluR5 NAM, showed target engagement with prepulse inhibition in participants with FXS [65], yet subsequent trials failed to show a behavioral benefit across the trial cohort in phase 2a and b trials in adolescents and adults [66,67]. This suggests that the preclinical data from models are not helpful in choosing agents for human trials or that the trials may not have been targeting the correct outcome at the appropriate age for a developmental disorder. Because the mGluR5 NAMs have synaptic and learning effects in the animal models not measured in the human trials, the ability of these drugs to affect learning and cognition in FXS has not yet been truly tested.

In order to determine if this class of drugs can help to accelerate learning in FXS, a novel trial is currently enrolling 3–6-year-old children with FXS in a double-blind placebo-controlled trial, in which subjects are randomized to AFQ056 or placebo and then receive a 6-month intensive language intervention to assess whether language learning can be enhanced by the drug. This study is also using a novel objective videotaped measure of communication during play tasks to assess improvements in language and communication, as well as standard objective measures of language and cognition, and eye tracking and auditory event related potential biomarkers, tests which should not be prone to placebo effects. This trial is being conducted through a Novartis independent investigator program project in partnership with an NIH grant to run the trial through the NIH-funded NeuroNext network as an exploratory new trial design to test targets in neurodevelopmental disorders with strong preclinical effects on synaptic and learning function in model systems. 

## 5. Considerations for Future Preclinical Research

The failure of animal models to predict the efficacy of potential therapies in psychiatric diseases and neurologic disorders, including FXS, is a central problem in drug development. The reproducibility of preclinical results remains a critical issue for translation. The industry has the resources to validate published results rapidly, but the issues with reproducibility of published results from one lab to another often hinder efforts and have a net impact of slowing the speed of drug development. Experience with the *Fmr1* KO model in predicting clinical success has demonstrated their value as experimental systems for proof-of-principle assessments of new interventions. However, this work has also shown that phenotypes that are improved in mice do not necessarily translate directly onto affected individuals. The translation of behaviors is especially difficult, and indeed, the available preclinical data suggest that the behavioral phenotypes in *Fmr1* KO mice do not translate well to behavioral symptoms measured in clinical trials.

In terms of progress, there are now electrophysiological measures that show similar abnormalities in *Fmr1* KO mice and individuals with FXS. Therefore, the field needs to emphasize the development of preclinical animal testing that can be evaluated in a similar manner in humans.

These markers need to be employed to help with target engagement and show direct translation from mouse to man. Furthermore, it would strengthen preclinical work to systematically validate the data across multiple assays from behavioral to electrophysiological to molecular, in at least two labs and in multiple species as per the NIH’s published guidelines.

Another barrier to translatability stems from the premature publication of results that haven’t been validated, and a publication culture focused on “positive” preclinical data. Understanding which particular treatments may not improve animal phenotypes may be as important as understanding the aspects of success with a specific drug. Thus, the industry would encourage the publication of negative preclinical data. These “negative” results would be critical to inform study design, outcome measure selection, and execution of future such studies in humans.

## 6. Considerations for Conducting Clinical Trials

As previous experience has shown, clinical trials to evaluate potential FXS pharmacologic treatments are challenging. From an industry perspective, there is much to be learned from previous clinical study failures, but also a wealth of resources upon which to draw in order to improve the study implementation/execution to increase the likelihood of developing safe and effective targeted treatment options for the FXS population.

Patient advocacy groups are uniquely positioned to provide insight into the needs of individuals with FXS and their families/caregivers, which can then be incorporated into trial design. Moving forward, it is important that the pharmaceutical industry collaborate with organizations such as the National Fragile X Foundation (NFXF) (www.fragilex.org) and FRAXA (www.fraxa.org) to broaden our collective understanding of outcomes that are pertinent and important to this community. Specifically, such relationships will help to ensure outcomes are relevant and clinically meaningful. Additionally, partnerships will help with recruitment and engagement in trials; an alliance with patient registries (e.g., the Fragile X Research Registry (www.fragilexregistry.org)) and the Fragile X Online Registry with Accessible Research Database (FORWARD) can also be considered as a mechanism to identify individuals and families affected by FXS that may be interested in study participation.

The industry must also reach out to the scientific community, which collectively harbors a wealth of knowledge from decades of research and clinical experience. It is notable that the US-based FRAXA Research Foundation (www.fraxa.org) not only funds research grants and fellowships at top universities, but also partners with biomedical and pharmaceutical companies to “bridge the gap between research discoveries and actual treatments”.

With respect to study design, some key considerations include the heterogeneity of the condition and the lack of sensitive, validated clinical outcome measures. For example, in a 2017 commentary published in *Translational Neuroscience*, Duy and Budimirovic [68] highlighted the need to utilize strategies to detect potential differences in response based on both heterogeneity of phenotype and baseline symptom severity. Additionally, outcome measures need to be able to capture the wide range of symptom presentations in the syndrome, and they need to be sensitive enough to detect potentially small changes, as in FXS, even small gains can be quite meaningful and impactful on individuals’ daily lives.

Desirable attributes of clinical outcome assessment measures are summarized in Table 3. Unfortunately, no reliable clinical outcomes’ assessment measures that fulfill these criteria have been established. In 2017, an expert working group review of tools used to quantify outcomes in FXS clinical trials did identify several promising measures, including the KiTAP [69,70], http://www.psytest.net/index.php?page=KiTAP, Expressive Language Sampling (ELS) [71], and the NIH-TCB [72,73]; (http://www.healthmeasures.net/explore-measurement-systems/nih-toolbox) for assessing cognition [34]; independent living scales (ILS; https://www.pearsonclinical.com/therapy/products/100000181/independent-living-scales-ils.html) and the Waisman activities of daily living scale (W-ADL; www.waisman.wisc.edu/family/WADL) [74] for evaluating adaptive behavior [34]; and the fragile X syndrome rating scale (FXSRS) for evaluating changes in behavior and emotion [34]. Additional studies, such as the ongoing SKYROCKET trial (http:/www.ovidrx.com/our-pipeline/)—a nondrug study of males with FXS aged 5 to 30 years examining the suitability of scales for the measurement of behavior, sleep, and functioning in individuals with FXS (OVID website)—are important to validate the appropriateness of these measures for inclusion in future FXS treatment trials.

Data quality and accuracy, overall, also need to be specifically considered in FXS trials. Given the nature of the condition, caregiver-report measures are critical to capture information about the subjects’ emotional and behavioral presentation, particularly. Many mothers of children with FXS tend to be generally anxious [75], in part related to the high likelihood of being a premutation carrier, and potentially caring for more than one child with FXS, and this heightened general anxiety may affect their ability to objectively recall and report on their child’s presentation. Therefore, training on how to complete measures accurately is of critical importance. Caregivers need direct instructions, clear examples, and concrete and specific anchors for reporting on behavior change. Similarly, in multisite trials, rater training is of the utmost importance. As with supporting caregivers in accurate reporting, trials need to provide instructions for clinicians on how to ensure reliability across raters and sites. Examples include a strong study-specific training curriculum, ongoing data quality assurances, and the use of objective outcomes when possible. For more subjective outcome assessments that rely on clinical judgment, trials need to employ highly expert raters and utilize training methods to ensure the clinicians are reliably and uniformly coding the subjects’ presentations.

In 2006, the NFXF brought together the network of majority of Fragile X clinics in the US into a “Fragile X Clinical and Research Consortium (FXCRC)”. The Foundation provides its administrative structure, such as meeting planning, material development, publicity, and internal communication. The Consortium’s members led by the Clinical Committee have written expert-level consensus documents (i.e., ASD, pharmacological, toileting, medical issues, etc.) primarily aimed at properly informing and empowering care providers and families of individuals with *FMR1* mutations. The Clinical Trials Committee (CTC) was formed in 2015 to try to help guide the industry with trial design and drug development planning to optimize the process and ensure stakeholder input. The CTC consists of the top clinical investigators in the FXS field who donate time to provide free consultation and input on any interventional trial to be performed on patients with FXS.

To integrate the work of basic and clinical intramural scientists and to facilitate progress in clinical trials, the NIH established a Bench-to-Bedside program in 1999. The program has continued to grow and in 2006, it opened to partnerships between intramural and extramural programs, which further enabled fruitful collaborations; for example, the investigation of signaling pathways of antipsychotics, which are widely prescribed in individuals with FXS [18].

Due to the intellectual disabilities in the FXS population, the clinical trial endpoints are heavily based on caregiver reports. These questionnaires are performed by caregivers based on their perception on the subject’s behavior, and these are highly susceptible to large placebo effects. The placebo response in FXS clinical trials is strong, as is the case in other CNS indications, and this may certainly contribute to the failure of many past studies to show a drug effect. Clinical trials in FXS often fail to show a statistically significant difference between the treatment and placebo control groups. In these cases, it is unclear whether the treatment was truly not effective or whether the larger than expected placebo response masked the treatment effect. The presence of a significant placebo effect makes it more difficult to observe a treatment effect because the treatment will need to result in a much larger improvement in order to see a positive response. Thus, it is important to include assessments that are less susceptible to placebo responses, such as direct subject assessments of cognition and language [18], and not rely on caregiver assessments alone.

At present, behavioral and cognitive measures are the most directly clinically meaningful outcome assessments. As reviewed in Budimirovic and colleagues (2017) [34], however, biomarkers are also critical to consider in clinical trials. Examples of biomarkers in FXS include blood and tissue markers (e.g., mitogen-activated protein kinases/extracellular signal regulated kinases (MAPK/ERKs), the brain-derived neurotrophic factor (BDNF), the amyloid precursor protein (APP)), neurophysiological measures (e.g., prepulse inhibition (PPI), eye tracking and pupillometry, event-related potentials (ERPs), electroencephalography (EEG) spectral analyses), and neuroimaging. As the field progresses, biomarkers will likely provide evidence for target engagement and possibly will serve as early efficacy indicators. Changes in neural signals or neurophysiological measures, for example, may be able to serve as indicators of treatment response prior to overt or measurable change in behaviors. Additionally, biomarkers would ideally be able to provide objective measures of treatment response as well as predictors of outcome. Given the heterogeneity in FXS despite the common etiology of the expansion mutation in 99% of cases, biomarkers may even be able to inform novel and personalized therapeutics and approaches. Further work, though, needs to be completed to both identify and validate potential biomarkers. At present, measurements of biomarkers tend to have limited feasibility due to cost, availability of equipment, and subjects’ ability to tolerate the procedures. Therefore, overall, the benefits of biomarkers on investigational FXS therapies remain to be established [34], but there are significant potential benefits of the approach, as the field progresses.

## 7. Conclusions

There remains a great need for safe and effective treatments for FXS, particularly for targeted treatments that surpass symptom management and address the pathophysiologic abnormalities that underlie the most common manifestations. The pharmaceutical industry can potentially aid this effort by taking on a more prominent role in both the preclinical and clinical phases of FXS drug development. It is imperative that the industry collaborate with both the research and advocacy communities to develop well-designed clinical studies that can produce meaningful results. This effort should include a leading role in the identification and validation of practical and reliable clinical outcomes assessments and biomarkers for use in future drug evaluation studies.

## Figures and Tables

**Table 1:** Common features of fragile X syndrome (FXS).

Neurocognitive [8]	Developmental Delays (Motor and/or Language) Cognitive Deficits/Intellectual Disabilities
Behavioral [8]	Hand flapping and/or biting Gaze avoidance Tactile defensiveness Hyperarousal to sensory stimuli Impaired social skills Social anxiety and mood disorders Hyperactivity Impulsivity Aggression Perseverative behavior
Physical Features [8,9]	Large ears Long, narrow face Prominent forehead or chin Large testicles in teen/adults High palate Flat feet Hyperflexible joints
Other [9]	Recurrent otitis media Strabismus Sleep disorders Gastroesophageal reflux Seizures Weight gain

**Table 2:** Ongoing* clinical trials of pharmacologic interventions for the treatment of FXS [35].

Phase	Compound	Identifier	Industry-Sponsored? (Y/N)
1	AZD7325	NCT03140813	N
2	Acamprosate, lovastatin, minocycline	NCT02998151	N
2	AFQ056	NCT02920892	N
2	BPN14770	NCT03569631	Y (Tetra Discovery Partners/FRAXA/RUMC)
2	Metformin	NCT03722290	N
2	OV101/gaboxadol	NCT03697161/ROCKET	Y (Ovid Therapeutics Inc.)
2/3	Acamprosate	NCT01911455	N
2/3	Metformin	NCT03479476	N
2/3	ZYN002	NCT03614663/CONNECT-FX	Y (Zynerba Pharmaceuticals Inc.)
4	Lovastatin	NCT02642653	N
4	Methylphenidate, fluoxetine, risperidone	NCT00768820	N

* Planned, recruiting/enrolling, or active; Y: Yes; N: No

**Table 3:** Desirable clinical outcome measure attributes [76].

Tests a broad range of ability Overcomes cooperation/variable performance problems Results can be reproduced Quantifies core defects Correlates with quality of life/true functional improvement
